# Mindful Reading: Mindfulness Meditation Helps Keep Readers with Dyslexia and ADHD on the Lexical Track

**DOI:** 10.3389/fpsyg.2016.00578

**Published:** 2016-05-10

**Authors:** Ricardo Tarrasch, Zohar Berman, Naama Friedmann

**Affiliations:** ^1^School of Education and Sagol School of Neuroscience, Tel Aviv UniversityTel Aviv, Israel; ^2^School of Psychology, Tel Aviv UniversityTel Aviv, Israel

**Keywords:** dyslexia, ADHD, MBSR, mindfulness meditation, attention, lexical route, surface dyslexia

## Abstract

This study explored the effects of a Mindfulness-Based Stress Reduction (MBSR) intervention on reading, attention, and psychological well-being among people with developmental dyslexia and/or attention deficits. Various types of dyslexia exist, characterized by different error types. We examined a question that has not been tested so far: which types of errors (and dyslexias) are affected by MBSR training. To do so, we tested, using an extensive battery of reading tests, whether each participant had dyslexia, and which errors types s/he makes, and then compared the rate of each error type before and after the MBSR workshop. We used a similar approach to attention disorders: we evaluated the participants’ sustained, selective, executive, and orienting of attention to assess whether they had attention-disorders, and if so, which functions were impaired. We then evaluated the effect of MBSR on each of the attention functions. Psychological measures including mindfulness, stress, reflection and rumination, lifesatisfaction, depression, anxiety, and sleep-disturbances were also evaluated. Nineteen Hebrew-readers completed a 2-month mindfulness workshop. The results showed that whereas reading errors of letter-migrations within and between words and vowelletter errors did not decrease following the workshop, most participants made fewer reading errors in general following the workshop, with a significant reduction of 19% from their original number of errors. This decrease mainly resulted from a decrease in errors that occur due to reading via the sublexical rather than the lexical route. It seems, therefore, that mindfulness helped reading by keeping the readers on the lexical route. This improvement in reading probably resulted from improved sustained attention: the reduction in sublexical reading was significant for the dyslexic participants who also had attention deficits, and there were significant correlations between reduced reading errors and decreases in impulsivity. Following the meditation workshop, the rate of commission errors decreased, indicating decreased impulsivity, and the variation in RTs in the CPT task decreased, indicating improved sustained attention. Significant improvements were obtained in participants’ mindfulness, perceived-stress, rumination, depression, state-anxiety, and sleep-disturbances. Correlations were also obtained between reading improvement and increased mindfulness following the workshop. Thus, whereas mindfulness training did not affect specific types of errors and did not improve dyslexia, it did affect the reading of adults with developmental dyslexia and ADHD, by helping them to stay on the straight path of the lexical route while reading. Thus, the reading improvement induced by mindfulness sheds light on the intricate relation between attention and reading. Mindfulness reduced impulsivity and improved sustained attention, and this, in turn, improved reading of adults with developmental dyslexia and ADHD, by helping them to read via the straight path of the lexical route.

## Introduction

In this study, we explore the unsolved riddle of the relation between attention and reading through a novel window: that of mindfulness meditation. We examine whether mindfulness meditation, which improves various aspects of attention, can have an effect on reading. We do so by exploring the effect of mindfulness on specific types of developmental dyslexia and on specific types of reading errors. The rationale is that if certain aspects of attention improve following mindfulness practice, and lead to improvement in certain aspects of reading, these aspects of reading may be related to attention. Furthermore, this may help define the conditions in which mindfulness can function as an effective treatment for reading difficulties.

### The Reading Process and Dyslexia

**FIGURE 1:**
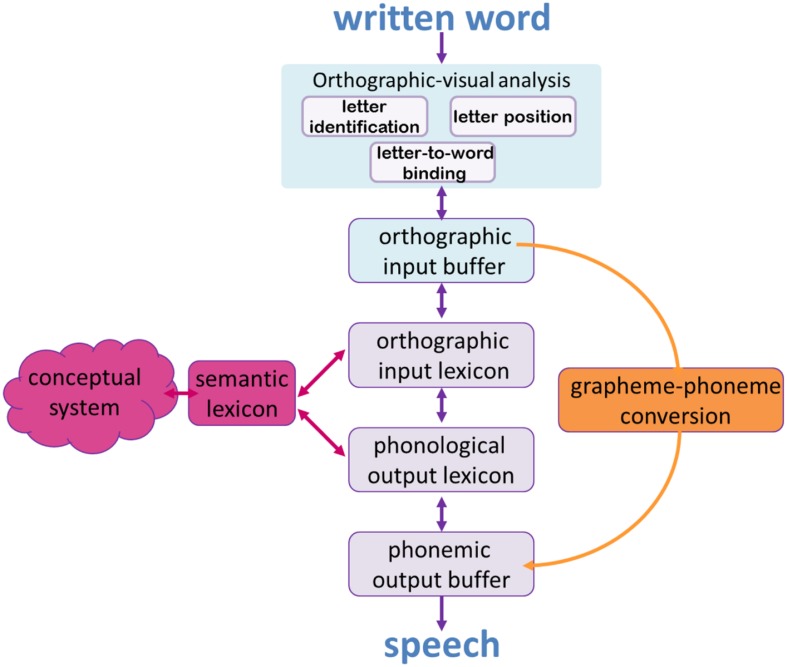
**The dual-route model for single word reading.** The straight, purple path denotes the lexical route, the orange path is the sublexical grapheme-to-phoneme conversion route.

The reading process is a multi-component process, which leads from the first orthographic-visual analysis of a written sequence of letters to sound and meaning. The model for single word reading that we assume here is the dual-route model (Morton and Patterson, 1980; Shallice and Warrington, 1980; Ellis and Young, 1996; Coltheart et al., 2001; Friedmann and Coltheart, in press). According to this model (depicted in **Figure 1**), the first stage of the process is a primary visual-orthographic analysis, in charge of letter identification, coding of the relative order of letters within the word, and binding letters to the words they appear in (Ellis and Young, 1996; Friedmann and Coltheart, in press). The results of this first visual analysis are then held in a short-term graphemic memory buffer, the graphemic input buffer.

Following this initial stage, the reading process divides into two routes: the lexical route, which allows efficient and rapid reading of words that the reader already knows, and which are stored in the orthographic input lexicon (the written form of the word), and the phonological output lexicon (its phonological form). Reading words that are not stored in these lexica requires the second route – the sublexical route, where reading is done via grapheme-to-phoneme conversion. This route is slower, and is inaccurate when reading words that do not unambiguously obey the grapheme-to-phoneme conversion rules.

Impairments in different stages and components of this process result in various types of dyslexia, each characterized by a different pattern of errors (Warrington and Shallice, 1980; Castles and Coltheart, 1993; Castles et al., 2006; Friedmann and Coltheart, in press). A deficit in letter identification causes *letter identity dyslexia*, which results in letter substitutions; a deficit in the encoding of letter position within a word causes *letter position dyslexia* (LPD, Friedmann and Gvion, 2001; Friedmann and Rahamim, 2007, 2014; Friedmann and Haddad-Hanna, 2012, 2014; Kohnen et al., 2012; Kezilas et al., 2014), characterized by letter migrations within words (such as reading *clam* as *“calm,”* and *flies* as *“files”*); a deficit in the allocation of letters to words results in *attentional dyslexia*, characterized by migrations of letters between neighboring words (Shallice and Warrington, 1977; Davis and Coltheart, 2002; Mayall and Humphreys, 2002; Friedmann et al., 2010b); a deficit in the identity of letters on one of the sides of a word, resulting in letter omission, substitution, or additions, is called *neglect dyslexia* (Ellis et al., 1987; Vallar et al., 2010). Impairments in the lexical route result in *surface dyslexia* (Marshall and Newcombe, 1973; Coltheart et al., 1983; Coltheart and Funnell, 1987; Howard and Franklin, 1987; Castles and Coltheart, 1993, 1996; Temple, 1997; Friedmann and Lukov, 2008). Readers with this dyslexia cannot use the lexical route, and they therefore have to rely on the sublexical route for reading aloud, and read words through grapheme-to-phoneme conversion. In this case, reading will be slower than usual, and, importantly for the current study, also inaccurate: reading of irregular words, namely, of words that do not obey the grapheme-to-phoneme conversion rules, will be incorrect. For example, the word *walk* may be read with a pronounced *l*, the word *knight* may be read with pronounced *k* and *g*, and the word *shoe* may be read as “show.” Furthermore, words with ambiguous conversion from graphemes to phonemes may also suffer— a word like *bear* may be read like “beer,” and *now* may be sounded out as “know.”

A deficit in the sublexical route also gives rise to impaired reading: disordered grapheme-to-phoneme conversion results in a situation whereby the readers can only read words they already know and fail to read new words and non-words. This is called *phonological dyslexia* (Temple, 1997).

Recently, another type of dyslexia has been reported, which involves a selective impairment in the sublexical route, which specifically affects the reading of vowel letters, *vowel dyslexia*. Individuals with vowel dyslexia make migrations, substitutions, omissions, and additions of vowel letters when they read via the sublexical route, almost without errors in consonants (Khentov-Kraus and Friedmann, 2011)^1^

### What Do We Know about the Relation between Reading and Attention Disorders?

The question of the relation between reading in general, and specific dyslexias on the one hand, and attention on the other is still an open one.

Epidemiological studies among children (Hinshaw, 1992) and adolescents (Hari et al., 1999, 2001; Goldston et al., 2007) report co-occurrence of diagnoses of ADHD and reading difficulties, and increased occurrence of poor reading in individuals with ADHD (August and Garfinkel, 1990; Dykman and Ackerman, 1991; Shalev-Mevorach et al., 2011). Some studies show poorer reading performance in the ADHD group in comparison to control participants without ADHD (Brock and Knapp, 1996; Miller et al., 2013). These findings are in line with the growing recognition that attention plays a crucial role in fluent reading (e.g., Cestnick and Coltheart, 1999; Reynolds and Besner, 2006; Shaywitz and Shaywitz, 2008; Ruffino et al., 2010). Recently, studies have shed more light on the relationships between specific attention functions and reading in skilled and dyslexic readers, emphasizing correlations between selective (Facoetti et al., 2006, 2010; Menghini et al., 2010), sustained (Menghini et al., 2010), and executive (Shalev-Mevorach et al., 2011) attention functions and successful reading.

However, the exact relation and mechanism of causality between ADHD and reading is not clear: text reading, for example, is a task that requires multiple skills, and many of them may be affected by attention. Performance in word and non-word reading tasks may also be affected by multiple factors. Furthermore, these studies have not examined the effect of attention on specific types of dyslexia or on specific types of errors in reading. As we have seen above, various types of dyslexias exist. Some of them may be thought to be associated with specific impairments to attentional processes: LPD, where letter migrations within words could result from inattention to middle letters; attentional dyslexia, where a difficulty in attenuating irrelevant neighboring words may be the cause for letter migrations between them; and neglect dyslexia, where attention allocating to one side of word or text may be the source for the neglect of that side in reading (see discussion in Lukov et al., 2015). However, various findings suggest that these dyslexias do not stem from attention disorders. Lukov et al. (2015) examined the relation between attention deficits and developmental LPD, attentional dyslexia, neglect dyslexia, surface dyslexia, and vowel dyslexia by systematically looking for dissociations between these dyslexia types and specific attention difficulties. They reported on 110 individuals who showed clear dissociations and double dissociations between these dyslexias and attention disorders, suggesting that attention disorders do not underlie these dyslexias.

Additionally, several studies found dissociations between the reading of words and that of numbers or symbols. Individuals with developmental neglect dyslexia who made neglect errors on the left side of words did not make errors on the left side of numbers (Friedmann and Nachman-Katz, 2004; Nachman-Katz and Friedmann, 2008). In addition, individuals with LPD, who made a considerable amount of transpositions of letters within words did not make more migrations of digits in multi-digit numbers compared to the normal rate (Friedmann et al., 2010a). In the same vein, adults with developmental dyslexia characterized by letter position errors and migration between words (which characterize LPD and attentional dyslexia, respectively) did not perform such errors among symbol strings (Collis et al., 2013). Had the reading errors in LPD, neglect dyslexia, or attentional dyslexia resulted from a general visuo-spatial attention deficit, we would expect this attention deficit to affect number reading as well. The findings that numbers are unimpaired in these dyslexias indicates that general visuo-spatial attention disorder does not underlie these dyslexias.

Relatedly, Salner et al. (2013) tested the effect of manipulations of spatial attention on letter transpositions in individuals with LPD using a Posner (cost-benefit) task. They found that the attentional manipulation did not affect letter transpositions. This result further corroborates the conclusion that LPD may reflect a specific impairment in letter position encoding, rather than a general attentional deficit.

Finally, Keidar and Friedmann (2011) found that methyl phenidate, which is the most commonly prescribed drug for treating ADHD, relieved attentional deficits, but did not affect reading accuracy among individuals with ADHD and developmental dyslexia (all with LPD and many with attentional dyslexia). These findings further support the idea that these types of dyslexia are orthographic-specific and are not the result of a general attentional deficit.

Thus, whereas there are some indications for points of connection and disconnection between attention and reading, the exact relation and mechanism of causality between them are still an open question. By assessing the effect of mindfulness on specific attention functions and specific error types in reading we hope to learn more about the nature of the relation between reading and attention.

### Mindfulness

A promising direction for the treatment of attention difficulties and possibly also of reading difficulties is the practice of mindfulness meditation. Among mindfulness practices, one of the most studied protocols is Mindfulness-Based Stress Reduction (MBSR). This protocol, developed by Kabat-Zinn (1990), is a well-established clinically oriented group-based meditation program, which has been widely used in the last few decades in various contexts. The foundation of MBSR is mindfulness meditation, defined operationally as “the awareness that emerges through paying attention on purpose, in the present moment, and non-judgmentally to the unfolding of experience moment by moment” (Kabat-Zinn, 2003). Mindfulness training has been shown to be beneficial for clinical and non-clinical populations, causing a decrease in anxiety (Kabat-Zinn et al., 1992), depression (Kumar et al., 2008), stress (Chiesa and Serretti, 2009), avoidance and rumination (Kumar et al., 2008), cognitive reactivity (Raes et al., 2009), and sleep disturbances (Carlson and Garland, 2005; Winbush et al., 2007), among others.

Importantly, mindfulness has a strong conceptual relation with attention, as a fundamental aspect of mindfulness practice is attentional training, and the role of attention is being emphasized in mindfulness instructions (e.g., Jha et al., 2007). Mindfulness practice has been shown to enhance attentional functioning, including sustained, selective, and executive attention (e.g., Valentine and Sweet, 1999; Wenk-Sormaz, 2005; Jha et al., 2007; Zylowska et al., 2008; Hodgins and Adair, 2010), however, some studies did not find such effects (McMillan et al., 2002; Heeren et al., 2009; MacCoon et al., 2014). Mindfulness was also found to improve other cognitive abilities, such as working memory (Chambers et al., 2008; Zeidan et al., 2010; Mrazek et al., 2013). Recently, Mrazek et al. (2013) reported undergraduate students to significantly increase their GRE reading comprehension scores following a 2-week mindfulness training program. The effects of mindfulness have also been reported to influence brain function (e.g., Davidson et al., 2003; Creswell et al., 2007; Goldin and Gross, 2010).

The positive effect of mindfulness on psychological measures can be helpful also in the context of people with dyslexia. During school years, reading deficiencies are often associated with embarrassment, frustration, lack of motivation and low self-esteem (Maughan, 1995; McNulty, 2003). Furthermore, recent data indicate that individuals with poor reading suffer from higher rates of psychiatric disorders, including anxiety and affective disorders (the rates of anxiety disorders, especially social phobia and generalized anxiety disorder, are significantly higher even when accounting for the presence of ADHD, Goldston et al., 2007). Therefore, it would be important to examine whether MBSR can relieve these effects in dyslexic individuals.

Combining existing knowledge on the role of attention in reading with the accumulating evidence of enhanced attention following mindfulness practice, we hypothesized that mindfulness can be used to improve reading among people with dyslexia. Moreover, the reported positive effects of mindfulness on other cognitive abilities as well as on practitioners’ wellbeing further suggest that this technique can serve as a highly beneficial intervention for this population. Thus, the aim of the present study was to investigate the effects of MBSR training on reading, attention, and psychological wellbeing among individuals with dyslexia and/or attention deficits. Specifically, we will ask whether reading errors decrease following mindfulness practice, whether specific types of reading errors are differentially affected by mindfulness, and which attention functions are sensitive to mindfulness practice. We will also explore the correlations between the effects of mindfulness on reading errors and on attention functions, to learn about the mechanism that ties reading and attention.

## Materials and Methods

### Participants

Twenty-four adults started the MBSR workshop. Among them, 17 participants had dyslexia and 14 had ADHD (seven participants had both dyslexia and ADHD). The participants were students in diverse academic fields. They were recruited in various ways: some were approached through the students’ dean’s office, who are in contact with students with learning disabilities, others were recruited via flyers that were posted in Tel Aviv University, inviting individuals with dyslexia and/or ADHD to participate in the study, and some were former participants of the lab’s studies who were invited to participate in this study.

The participants paid a symbolic fee for participating in the MBSR workshop, and committed to attend the meetings and exercise the workshop’s tasks at home. Twenty participants completed the workshop. One participant was excluded from the analyses due to a history of stroke. None of the other participants had a history of brain injuries or neurological problems. The final group of participants included 12 students with dyslexia and 13 with ADHD (six had both). All participants had normal or corrected-to-normal vision. Main demographic characteristics of the participants are presented in **Table 1.**

**Table 1:** Main demographic characteristics of experimental and control groups.

Age mean (SD)	30.6 (5)
Gender	10 females16 males
Handedness	4 left, 22 right
Mother tongue	23 Hebrew, 1 Hebrew and English, 1 English, 1 Russian
Years of education mean (SD)	15.2 (1.4)

The study was approved by the Tel Aviv University ethics committee, and written informed consents were obtained from all participants prior to the testing.

In order to determine the reading and attention profiles, we administered to each of the MBSR participants a battery of reading, attention and psychological tests, which we describe below.

### Materials and Procedure

#### Reading Assessment

For the initial assessment of reading, we used the TILTAN screening test (Friedmann and Gvion, 2003). This test uses oral reading of 128 single words, 30 word pairs, and 30 non-words. The word list in the screening test included words of various types that can reveal the different types of dyslexia: 67 migratable words – words in which middle letter migration creates another existing word, for the identification of LPD; 104 words for which omission, substitution, migration, or addition of a vowel letter creates another existing word, for the identification of vowel letter dyslexia; 128 words for which neglect of the left side of the word yields another existing word, for the identification of neglect dyslexia, and 108 words for which right neglect errors create an existing word; 84 irregular words and potentiophones for the identification of surface dyslexia (notice that given that in Hebrew there are no words that can be unambiguously converted to a single phonological string via grapheme-to-phoneme conversion, in fact all words in the test were sensitive to surface dyslexia); 57 morphologically complex words for deep dyslexia and phonological dyslexia; and 26 abstract nouns and 28 function words, for deep dyslexia. All the words were sensitive to visual dyslexia, as each word had more than six orthographic neighbors.

The 30 non-words were included for the identification of impairments in the sublexical route, phonological dyslexia, vowel dyslexia, and deep dyslexia, but also contained migratable non-words and non-words that created existing words by substitution, omission, or addition of letters, and were hence also sensitive to various impairments at the orthographic-visual analyzer (visual dyslexia, neglect dyslexia). The list of 30 word pairs was created so that between-word migrations created other existing words, for the identification of attentional dyslexia.

On the basis of this test, we determined, according to a comparison of the error rate of each participant to an age-matched control group of 372 skilled readers, whether she or he had dyslexia. If the participants had dyslexia, we determined which type(s) of dyslexia they had, on the basis of the types of errors they made and the factors that affected their reading – the types of stimuli that were most prone to reading errors and the factors that affected their reading (frequency effect, word length effect, lexicality effect, etc.). This diagnosis of the type of dyslexia guided the additional tests that we administered to each participant, in order to further establish the dyslexia type.

For example, participants who made mainly regularizations errors in irregular words in the screening task were suspected to have surface dyslexia and were therefore administered the continuation tests for surface dyslexia (see **Appendix A**); individuals who made a significant rate of letter transpositions within words in the screening task were further tested with the LPD tests, etc.

The performance of each of the participants in the screening test and in each of the reading tests was compared with the performance of a control group (372 adults) that was tested throughout the development of the test batteries. Each participant’s performance was compared to the control group using the Crawford and Howell’s (1998) *t*-test for the comparison of the performance of a participant with a control group. An impaired performance was defined as performance that was significantly below the control, with *p* < 0.05. The type of dyslexia was determined using the same procedure and statistical test, applied to each error type. We determined that a participant had a certain dyslexia if s/he made significantly more errors of the relevant type compared to the control group, and performed significantly poorer than the control group in the relevant reading tests. We only included individuals in the no-dyslexia group if they performed within the normal range in all the reading tests.

**Letter position dyslexia** was determined according to the number of letter position errors (consonant migrations) in reading migratable words; **attentional dyslexia** was determined according to the number of between-word errors, including between-word migrations and between-word letter omissions, in reading migratable word pairs; **surface dyslexia** was determined according to the number of reading errors that resulted from reading via the sublexical route rather than via the lexical route, which caused regularization errors in irregular words and potentiophones; **vowel dyslexia** was determined according to the number of vowel letter errors (migrations, substitutions, omissions, and additions) in words and non-words; and **phonological dyslexia** was determined according to a significantly larger number of errors (substitutions, omissions, and additions) in non-words compared to words. See **Appendix A** for details on the tests that we administered to further establish the diagnosis of each type of dyslexia.

The analyses of error rates before and after the workshop were done for each participant out of the tests that s/he did, according to their dyslexia.

*Surface dyslexia errors* were analyzed from the screening test as well as an additional task of potentiophone reading, in which the participant read aloud 78 potentiophonic words, 2–6 letters long (mean = 3.7 letters, *SD* 0.8). Potentiophones are words whose reading via grapheme-to-phoneme conversion creates another existing word (like *now*, which can be read via grapheme-to-phoneme conversion to sound like “know,” Friedmann and Lukov, 2008). Such words are the most sensitive stimuli to detect surface dyslexia because when a person reads them via the sublexical route, s/he does not know that the word was read erroneously, because another word was produced.

*Letter position errors* were calculated out of the migratable words that the participant read in the screening test and in an additional test of reading aloud of 232 migratable words of 4–7 letters (mean = 4.9, *SD* 0.9). Migratable words are words for which migration of middle letters within the words creates another existing word (such as *bread-beard, sings-signs*); 87 of these words had a lexical potential for a migration that involves a vowel letter, and 163 had a potential for a migration that involves only consonant letters. (For an English example, the word *sings* has a potential for transposition of two consonant letters- *n* and *g*, whereas the word *snag* has a potential for migration that involves a vowel – a transposition of *a* and *n*.)

*Migrations between words* were calculated out of the word pairs in the screening test as well as the additional task of reading aloud of 120 migratable word pairs of 2–7 letters (mean = 4.8, *SD* 1.0). All the word pairs were migratable, namely, for each of them, migration of a letter from one word to the other, preserving the within-word position, creates another existing word (such as *mild wind* in which between-word migration can create *wild mind*).

*Vowel letter errors* were calculated out of the non-words in the screening test as well as a test of reading aloud of 60 non-words, 3- to 6-letter long (mean = 4.45, *SD* 0.67). The non-word list was constructed so that for each non-word at least two different vowel errors would create existing words (a parallel example in English would be the non-word *bron*, which, with a vowel error could be read as *born*, *bran*, and *baron*.)

Each individual was tested separately, in a quiet room, and the reading tests were administered with no time limit. The resulting diagnoses of the participants are presented in **Table 2.**

**Table 2:** Diagnoses of reading and attention skills of MBSR participants and indicator of completion of workshop.

Participant	Diagnosis		Completion of workshop
	Dyslexia type(s)	Attention deficit(s)	
AO	LPD, attentional, surface	–	Yes
ALE	LPD, surface, attentional, vowel	–	No
EZ	–	Sustained attention	Yes
NG	LPD	–	Yes
ON	–	Sustained attention	Yes
YH	LPD, surface, attentional	–	No
SG	LPD, attentional, vowel, surface	Sustained attention	Yes
RC	Vowel	Sustained attention	Yes
YS	–	Orienting of attention, executive attention	Yes
RA	LPD, attentional	–	Yes
GA	LPD, surface	Sustained attention	Yes
OR	LPD, vowel, attentional, surface	Sustained attention	Yes
AF	LPD	–	Yes
TL	–	Sustained attention, orienting of attention	Yes
ET	LPD, vowel, surface	–	Yes
RS	Vowel	–	Yes
RB	LPD, vowel, attentional, surface	–	No
GS	Vowel, LPD, surface	Sustained attention	Yes
YK	LPD, surface	Selective attention	Yes
JO	–	Orienting of attention	Yes
ALU	Attentional, LPD	–	No
SY	–	Sustained attention	Yes
MS	–	Sustained-, selective-, executive- and orienting of attention	Yes
OT	Phonological	Sustained attention	No

#### Attention Assessment

We used four attention tasks that were developed to test each of the four functions of attention model, proposed by Tsal et al. (2005) in the context of ADHD. The model includes: *(a) sustained attention* – the ability to allocate attentional resources to a non-attractive task over time while maintaining a constant level of performance; *(b) selective (spatial) attention* – the ability to focus attention on a relevant target while ignoring adjacent distractors; *(c) orienting of attention* – the ability to direct attention over the visual or auditory field according to sensory input, and to disengage and reorient efficiently; and *(d) executive attention* – the ability to resolve conflicts of information and/or responses. We assessed the four functions of attention by using four computerized neuropsychological tasks, serving as indicators of performance in each of the attention functions. All four tasks were established by Tsal et al. (2005), where they were used to assess attention functioning in children with and without ADHD. Each attention test started with a short practice block and lasted approximately 12 min.

For *sustained attention*, we used a Continuous Performance Test (CPT). Participants were presented with a long series of stimuli and were instructed to respond to a single reoccurring pre-specified target (a red square) while withholding responses to all other, non-target stimuli. There were four possible shapes (square, circle, triangle, and star) and four possible colors (red, blue, green, and yellow). As soon as a target appeared, the participants were requested to press the spacebar. Using a low rate of target stimuli (30%) and varying the inter-stimulus interval (ISI) with an average ISI of 1750 ms, this task maintains a high demand on sustained attention but minimizes the involvement of other cognitive factors (Shalev et al., 2011; Stern and Shalev, 2013). The standard deviation of the participant’s reaction times (RTs) indicates her/his ability to consistently maintain attention to the task over time. Thus, low standard deviation of RTs reflects high level of sustained attention, whereas a high standard deviation indicates inattention. The percent of commissions (responses to trial without target) depicts impulsivity.

For *selective attention*, we used a conjunctive search task (Tsal et al., 2005). Participants were instructed to search for a target stimuli appearing among distracters. The displays varied in their size (4, 8, 16, or 32 distractors), enabling estimation of the effect of attentional load on performance. Participants were instructed to fixate on a fixation point, which was followed by a display of items. Participants were requested to decide whether the display contained the target – a blue square – among the distractors (blue circles and red squares). The target appeared in 50% of the displays. If a target was detected, the participant had to press the ‘L’ key in the computer’s keyboard and if the target was absent then s/he had to press the ‘A’ key. To assess performance on this task both RTs and accuracy rates were recorded; however, selective attention is usually represented in this kind of task by the slope of the search function. Thus, the performance measure was defined as the search slope. This measure reflects the efficiency of the search process and is based on the increase in response time and decrease in accuracy observed as a function of the increase in search load – the number of stimuli (geometric shapes) in each cluster. This measure is extracted for target-present trials only.

For *orienting of attention*, we used a cost-benefit paradigm with peripheral cueing (Posner et al., 1980) with an exogenous cue (Jonides, 1981). Participants had to discriminate a stimulus – a triangle or a circle – (at either the left or the right of a fixation point) preceded by an abrupt onset of a cue at either the target’s location (a highlighted rectangle enclosing the stimulus – valid cue) or the opposite side of fixation (invalid cue). When the target was a triangle, the participant had to press the ‘L’ key and when the target was a circle s/he had to press the ‘A’ key. Both RTs and accuracy rates were recorded, and the difference in performance between valid and invalid trials was used to indicate the ability to efficiently orient attention (Tsal et al., 2005).

For *executive attention*, we used a Location-Direction Stroop-like task (Stroop, 1935) with a spatial aspect. Participants had to respond either to the location or the direction of an arrow (in different blocks) appearing on the screen, while ignoring the other irrelevant dimension. Half of the stimuli were congruent trials (i.e., the location on the screen and the direction of the arrow matched; e.g., an arrow presented below fixation pointing downward) and half of them were incongruent (i.e., an arrow presented above fixation pointing downward). In the first two blocks of the task, the participants were requested to judge the location of the arrow (relative to the fixation point; if it was presented above the fixation they had to press ‘L’ and if it was presented below the fixation they had to press ‘A’) and in the last two blocks they were requested to judge its direction (Tsal et al., 2005). Performance in this task was assessed by mean RTs and accuracy rates, and by subtracting congruent RT divided by accuracy rate from incongruent RT divided by their accuracy rate. The widely used interference effect in this task reflects the extent to which conflicting irrelevant information is effectively suppressed. Due to technical failures, the post-workshop data is missing for three participants in executive attention, for two in orienting of attention, and for one in selective and sustained attention.

We analyzed each participant’s baseline performance in each of the attention tasks in order to determine the existence and nature of attention deficits. The resulting diagnoses of the participants are presented in **Table 2.**

#### Resistance of the Tools to Training Effects

Because we examine the performance before and after the meditation workshop using the exact same tools, we needed to establish that the tools we used were not sensitive to training effects. We therefore administered the reading and attention tests twice, 3 months apart, to a group of students who did not participate in the MBSR workshop. The students in this group had learning disabilities that were similar to the ones we examined in this study.

##### Reading tests: control group without MBSR

In the reading tests of the non-MBSR control group, out of 16 dyslexics (who had types of dyslexia that were similar to the MBSR dyslexia group: LPD, attentional dyslexia, vowel dyslexia, and surface dyslexia), nine made more errors in the second assessment than in the first assessment, while seven made fewer errors. The average error rate in the reading tests did not change [before 16.6% (*SD* 12.1%); after 16.6% (*SD* 13.6%)]. This was true also when analyzing words and non-words separately. For existing words the average error rate was 13.4% (*SD* 10.5) in the first assessment, and 13.2% (*SD* 11.1%) in the second, *p* = 0.86. The average error rate in reading non-words was 29.8% (*SD* 21.2) in the first assessment, and 30.6% (*SD* 25.7) in the second, *p* = 0.83. The consistency in the percent of errors over time also held in the analysis of surface errors alone (*p* = 0.93). Namely, neither general error rate nor surface errors benefitted from the repetition of the same reading tests after 3 months.

##### Attention tests: control group without MBSR

Eighteen participants in the control group performed the attention tests twice, without MBSR in the middle, two of them had missing data in the CPT test.

In **the sustained attention** test, no significant reduction was obtained in the standard deviation of reaction times (RTs) in the CPT task (first measure: 111.1 ms [*SD* 51.3], second measure: 100.7 ms [*SD* 46.9], *p* = 0.29). Overall, 69% of participants (11 out of 16) had larger standard deviations compared to the norm in this measure in the first assessment (>77 ms), and also in the second one. No significant difference was found in the measure of commissions between the two assessment points (first measure: 1.1% [*SD* 0.9%], second measure: 0.6% [*SD* 0.8%], *p* = 0.09).

For **selective attention**, repeated measures ANOVAs with the within-subjects factors of time (first/second measurement), target (with/without target), and number of distracters (4, 8, 16, or 32) in the conjunctive search task, were performed separately for RTs and accuracy. The analyses yielded only a significant effect of time in RTs (first measure: 934.1 ms [*SE* 53.6], second measure: 854.4 ms [*SE* 39.6], *F*(1,17) = 9.52, *p* = 0.01, $\eta_{p}^{2}=0.36$). No significant time effect was obtained in accuracy (*p* = 0.97), and no significant differences were obtained for the search slope (*p* = 0.42).

For **orienting of attention**, two repeated measures ANOVA were performed with the within-subjects factors of time (first/second measurement) and target (valid/invalid cue) in the peripheral cueing paradigm, one for RTs and one for accuracy. An approaching significance reduction was found in RTs (first measurement: 667.0 ms [*SE* 46.5], second measurement: 595.2 ms [*SE* 18.8], *F*(1,17) = 4.3, *p* = 0.054, $\eta_{p}^{2}=0.20$). No significant overall improvement in accuracy was obtained (*p* = 0.91). The difference between valid and invalid conditions did not change significantly between the two time points (*p* = 0.97).

For **executive attention**, two repeated measures ANOVAs were performed with the within-subjects factors of time (first/second measurement), kind of task (location/direction) and congruency (congruent/incongruent) in the location-direction Stroop-like task. No significant differences in RTs nor in accuracy or interference were obtained (*p* = 0.86 and *p* = 0.88, respectively). The difference between the interference effect in the two time points was also not significant (*p* = 0.94).

Thus, in the attention tests too, the main attention measures (apart from RT) were unaffected by the repetition of the same tests after 3 months, without MBSR training in between.

#### Psychological Measures

Eight psychological domains were assessed among the MBSR participants using a battery of seven questionnaires. Participants filled out the questionnaires through an online site^2^ before and after the workshop, when they were available, at home, and free from other activities. The battery included the following questionnaires:

##### Mindfulness

This 26-item questionnaire was developed by Friedman (2006), based on two mindfulness questionnaires: the Mindful Attention Awareness Scale (MAAS; Brown and Ryan, 2003) and the Kentucky Inventory of Mindfulness Skills (KIMS; Baer et al., 2004). The questionnaire contains statements describing mindful and mindless experiences (e.g., “I tend to walk quickly to get where I’m going without paying attention to what I experience along the way”), each responded to on a 7-point scale, running from 1 (*not at all*) to 6 (*very much*). The original questionnaire has a Cronbach’s alpha of 0.79. In the present study, Cronbach’s alphas were 0.85 and 0.9 in the first and second administrations, respectively.

##### Perceived stress

The Perceived Stress Scale (PSS; Cohen et al., 1983) contains 14 items that describe emotions and feelings regarding stressful situations in one’s life (e.g., “In the last month, how often have you felt that you were unable to control the important things in your life?”). The participants were requested to respond on a 5-point scale ranging from 0 (*never*) to 4 (*very* often), according to their experience in the past month. The questionnaire is scored as the sum across all items, after reversing the positive ones. The instrument was translated to Hebrew by Drory (1989), and was reported to have an internal consistency of 0.77 in its Hebrew version. In the present study, this questionnaire had a Cronbach’s alpha of 0.9 and 0.91 in the first and second administration, respectively.

##### Reflection and rumination

The Rumination–Reflection Questionnaire (RRQ; Trapnell and Campbell, 1999) is a 24-item instrument consisting of two 12-item subscales: Reflection, addressing the degree of the person’s self-focusing that stems/derives from curiosity and interest in the self (e.g., “I love exploring my ‘inner’ self”); and Rumination, addressing the degree of the person’s self-focusing that stems/derives from threat, loss or injustice caused to her/him (e.g., “I spend a great deal of time thinking back over my embarrassing or disappointing moments”). Each item is responded on a 1 (*strongly disagree*) to 5 (*strongly agree*) scale. The questionnaire was translated to Hebrew by Margalit (2003). In the present study, the Reflection subscale had a Cronbach’s alpha of 0.93 in the first administration and an alpha of 0.92 in the second, while the Rumination subscale had a Cronbach’s alpha of 0.89 in the first administration and an alpha of 0.91 in the second.

##### Life satisfaction

The Satisfaction with Life Scale (SWLS; Diener et al., 1985) contains five statements referring to judgments of global satisfaction with one’s life (e.g., “so far I have gotten the important things I want in life”), each responded to on a 7-point scale ranging from 1 (*strongly disagree*) to 7 (*strongly agree*). The score was the items’ mean ratings. The English version was reported to have highly favorable psychometric properties, with a test–retest correlation coefficient of 0.82, an alpha coefficient of 0.87 and a mean correlation of 0.61 with other measurements of life satisfaction (Diener et al., 1985). The score was the items’ mean ratings. The questionnaire was translated to Hebrew by Shmotkin (P.C., 17.3.2011). In its translated version, the instrument’s alpha coefficient was reported to be 0.76 (Shmotkin and Lomranz, 1998). In the present study, this questionnaire had a Cronbach’s alpha of 0.84 in the first administration, and an alpha of 0.81 in the second.

##### Depression

The Center for Epidemiologic Studies – Depression Scale (CES-D scale; Radloff, 1977) is composed of 20 items (e.g., “I thought my life had been a failure”), each responded to regarding the degree of experiencing them in the past week on a 4-points scale running from 1 (*rarely or never [less than 1 day]*) to 4 (*most or all of the time [5–7 days]*). A test–retest reliability of the instrument was reported as 0.83 (Radloff, 1977). The questionnaire’s score was calculated as the respondent’s mean rating, with positive items reversed. The Hebrew version was reported to have a Cronbach’s alpha of 0.88 (Shmotkin et al., 2003). In the present study, this questionnaire had a Cronbach’s alpha of 0.9 in the first administration, and an alpha of 0.88 in the second.

##### Anxiety

The State-Trait Anxiety Inventory (STAI; Spielberger et al., 1970) was used to measure state anxiety. This scale contains 20 items (e.g., “I feel nervous”), each responded to on a 1 (*not at all*) to 4 (*very much*) scale, relating to the present moment. The state anxiety scale was reported to have a Cronbach’s alpha of 0.89 in its English version (Spielberger et al., 1970). The questionnaire was translated to Hebrew by Teichman and Melineck (1978), who reported the English and Hebrew versions to have correlations of 0.77–0.84 between them. In the present study, this questionnaire had a Cronbach’s alpha of 0.94 in the first administration, and an alpha of 0.95 in the second.

##### Sleep disturbances

The mini-Sleep Questionnaire was developed in Hebrew at the Technion Sleep Laboratory by Zomer et al. (1985). The questionnaire comprises 10 items tapping both insomnia and excessive daytime sleepiness. Each item is scored on a 7-point Likert scale ranging from 1 (*never*) to 7 (*always*). In the present study, this questionnaire had a Cronbach’s alpha of 0.81 in the first administration, and an alpha of 0.83 in the second.

### The MBSR Workshop

The Mindfulness-Based Stress Reduction (MBSR) is a structured group program developed by Kabat-Zinn (1990). According to its protocol, the participants attended eight weekly 2.5-hour classes and a half-day retreat with intensive practice during the sixth week. During these sessions, participants received training in mindfulness through body scan meditation, where participants bring attention to each body part, observe their sensations, and if pain or unpleasant sensations are felt, try to describe them objectively and then intentionally relax each body part; sitting meditation, where participants are instructed to sit in a relaxed, upright posture and to direct their full attention to the sensations of breathing, attending to and simply acknowledging any sensations that arise in the body, and non-judgmentally witnessing whatever thoughts arise, trying to merely label them and restoring attention to the breath; mindful stretching exercises, based on Hatha yoga, and mindful eating, where participants are instructed to be non-judgmental and fully aware, with all senses, of different aspects of the food that they eat (usually a raisin). The integration of mindfulness into everyday life and the application of mindfulness as a method for noticing habitual reactions to stressful situations and more creatively responding are greatly discussed in the group (Kabat-Zinn, 1990). In addition to the practice in the class, participants were asked to engage in mindfulness meditation practice for 45 min per day, guided by CDs and MP3 files that were provided. The workshop took place in a suitable auditorium and was instructed by a female clinical psychologist trained to teach MBSR by Jon Kabat-Zinn.

### Statistical Analyses

To assess the effects of the workshop on reading, attention skills, and psychological measures among the MBSR participants, all tests were administered twice to each participant, once prior to the workshop and once during the week after the workshop ended. To assess changes in reading ability, we first compared overall change in number of reading errors using a paired *t*-test. Next, to assess the effect of the workshop on the reading of each participant, we counted each participant’s number of errors typical of his/her dyslexia type/s (for example, migrations between words for participants with attentional dyslexia) made in the oral reading tests before and after the workshop, and calculated an improvement index for each participant as: (number of errors before – number of errors after)/number of errors before. Using this measure, we then tested the mean individual change using a paired *t*-test. Paired *t*-tests were also used for assessing changes in the main attention measures as well as for the psychological indexes. As sustained attention is the most prominent deficit among ADHD individuals, and in order to compare the improvement after the workshop between ADHD and non-ADHD participants, a repeated measurement ANOVA with the within-subjects factor of time of measurement and the between-subjects factor of ADHD diagnosis (present vs. absent) were performed on the standard deviation of RT’s. In order to assess changes in selective attention, orienting of attention, and executive attention, repeated-measures ANOVAs were performed. Only main effects of time of measurement (before vs. after the workshop) or interactions with it were reported and further explored, as other effects or interactions are related to general characteristics of the tasks and are not part of the scope of the present study (e.g., main effect of number of distracters in the conjunctive search task with longer reaction times on larger displays). Significant interactions were followed by Tukey honest significant differences (HSD) *post hoc* comparisons. *T*-tests are reported with means and standard deviations (in square parentheses), and repeated measures-ANOVAs are reported with means and standard errors. Finally, in order to assess whether the improvement in reading was correlated to improvements in attentional and psychological measures, Pearson correlations were calculated between the differences in these measures, calculated as: measure before the workshop – measure after the workshop. In order not to inflate the number of correlations calculated, only overall accuracy measures were used for the conjunctive search, cost-benefit, and Stroop-like tasks, and commissions percentage was used for the CPT task. Furthermore, we controlled for multiple tests type I error by applying the Benjamini and Hochberg (1995) false discovery rate (FDR) separately for the correlations of each type.

## Results

### Reading

#### The Effect of MBSR on the Whole Group of Participants with Dyslexia, and on Various Types of Reading Errors

Calculation of individual improvement indices revealed that most dyslexics (10 out of 12, χ^2^ = 5.33, *p* = 0.02) made fewer reading errors following the workshop, out of all the words that they read (6 of them significantly, McNemar’s tests, *p* < 0.01). The average error rate in all the reading tests decreased significantly from 12.7% (*SD* 6.4%) before the workshop to 9.7% (*SD* 4.5%) after it. This yielded a mean improvement of 3% (*SD* 3.9%), which formed 18.5% of the errors the group of dyslexic participants made before the workshop. This overall improvement was significant at the group level, *t*(11) = 2.60, *p* = 0.02, Cohen’s *d* = 0.75.

When analyzing words and non-words separately, this improvement was significant only for the existing words. Error rate in word reading decreased significantly from 12.1% (*SD* 6.4%) before the workshop to 9.2% (*SD* 4.5%) after it, yielding a mean improvement of 2.9% (*SD* 3.8%), *t*(11) = 2.65, *p* = 0.03, Cohen’s *d* = 0.76). The decrease in error rate in non-word reading was not significant (before: 18.0%, *SD* 15.8%, after: 14.3%, *SD* 10.9%, mean improvement 3.7%, *SD* 8.9%, *t*(11) = 1.43, *p* = 0.18).

Possibly the most important finding comes from the analysis of improvement separately for each type of error. When we analyze, for the group, the improvement in each type of error out of the relevant words for this error type, we see that surface errors showed a significant decrease following the MBSR training, and that they were the only type of error that showed this significant improvement (see **Table 3**). Namely, following meditation, the dyslexic participants read words more consistently via the lexical route rather than via grapheme-to-phoneme conversion. This finding is also consistent with the finding that the improvement was significant only for words but not for non-words: if the mindfulness workshop affected reading by leading readers to read words via the lexical, rather than the sublexical route, this is not expected to affect non-words, which are read exclusively via the sublexical route.

**Table 3:** Comparison of reading errors of the various types before and after the MBSR workshop for the whole group of dyslexic participants: average % (SD) of the various error types, before and after the MBSR workshop and *t*-test for dependent samples.

	% Errors before MBSR	% Errors after MBSR	Comparison before and after
All errors in words	12.1% (6.4%)	9.2% (4.5%)	*t*(11) = 2.65, *p* = 0.03
All errors in non-words	18.0% (15.8)	14.3% (10.9%)	*t*(11) = 1.43, *p* = 0.18
Surface errors	9.9% (7.9%)	6.9% (6.3%)	*t*(11) = 3.09, *p* = 0.01
Migrations within words	8.0% (3.1%)	6.1% (3.4%)	*t*(11) = 1.90, *p* = 0.08
Migrations between words	14.2% (12.3%)	12.1% (14.3%)	*t*(11) = 0.82, *p* = 0.43
Vowel letter errors in non-words	9.9% (10.4%)	7.6% (7.4%)	*t*(11) = 1.73, *p* = 0.11

*Shaded cells indicate a significant improvement between before and after the MBSR training.*

To examine our assumption that the reduction in reading errors is modulated by the effect of mindfulness on attention, we compared the effect of mindfulness on dyslexic participants who had ADHD (*n* = 6, all but one of them had sustained attention impairment) and dyslexic participants without ADHD (*n* = 6). We analyzed the reduction in the percent of reading errors separately for each sub-group. In spite of the small power of such tests, the results suggest that the reduction in reading errors stems from dyslexic participants who also had ADHD (Without ADHD a reduction from 10.7% [*SD* 5.7%] to 8.6% [*SD* 2.9%], *t*(5) = 1.16, *p* = 0.30; with ADHD a reduction from 14.7% [*SD* 6.9%] to 10.9% [*SD* 5.8%], *t*(5) = 2.71, *p* = 0.04). In line with the idea that the effect on the total number of errors is through improved ability to read via the lexical route, rather than occasional slipping to the sublexical route, the significant reduction in reading errors in the dyslexia+ADHD group was only present in reading words, *t*(5) = 2.92, *p* = 0.03, and was not present in reading non-words, *t*(5) = 1.57, *p* = 0.18.

#### The Effect of MBSR on Specific Dyslexia Types

To examine whether the MBSR training affected specific types of dyslexia, we examined, for each type of dyslexia that our participants had, the effect of MBSR on the relevant error type. The analyses were performed on the percentage of errors out of all possible errors that participants could have performed within the words they read. **Table 4** summarizes the results of the reading tests of each of the 12 participants with dyslexia who completed the workshop in words that target their specific type(s) of dyslexia before and after the workshop.

**Table 4:** Number of reading errors made by dyslexic participants before and after the workshop, separately for each error type; mean overall change per participant across types [(errors after – errors before)/(errors before)]; the number of words each participant read that could detect each error type (migratable words for migration errors, irregular words for surface errors, etc.); and total number of words read in all tests assessed at each timepoint.

Participant	Number of errors								Mean change of all errors (%)	Total number of words that are sensitive to errors relevant to the dyslexias diagnosed	Total number of words and non-words read
	LPD		Vowel		Attentional		Surface				
	Before	After	Before	After	Before	After	Before	After			
AO	50	25			44	27	16	7	–45%	658	698
NG	27	18							–8%	345	698
SG	17	29	15	17	12	16	47	33	2%	826	936
RC			12	2					–77%	90	464
RA	16	12			2	4			–24%	327	458
GA	35	22					54	33	–25%	586	776
OR	64	50	22	11	38	16	22	19	–35%	688	698
AF	19	32							–26%	297	458
ET	31	19	34	32			15	14	–31%	558	696
RS			19	10					–30%	90	618
GS	17	12	12	11			3	1	–6%	528	618
YK	21	22					20	24	85%	468	536

*Note that the overall change is based on the number of unique errors in all tests, which do not correspond to the sum of the four specific error types.*

*LPD: middle letter transposition within a word.*

*Vowel: substitution, omission, addition, or migration of a vowel letter.*

*Attentional: migration of a letter between words.*

*Surface: error resulting from reading via the sublexical route instead of the lexical route – phonologically plausible errors.*

This analysis did not yield any significant specific reduction of errors for any of the dyslexia types. The effect on LPD was assessed by the measure of reduction in letter migrations within words in reading 345 migratable words before and after the workshop for the 10 participants with LPD who participated in the workshop. This analysis yielded no significant reduction: reduction from 9.0% (*SD* 4.5%) to 7.4% (*SD* 3.3%), *t*(9) = 1.44, *p* = 0.18.

The effect on attentional dyslexia was assessed by the measure of reduction in letter migrations between words in reading 150 migratable word pairs before and after the workshop for the four participants with attentional dyslexia who participated in the workshop. This analysis also yielded a reduction from 17.3% migrations between words (*SD* 11.7%) to 13.2% (*SD* 3.5%), which was not significant, *t*(3) = 0.80, *p* = 0.48).

The analysis of the reduction of surface errors in surface dyslexia was made out of the phonologically legal reading of 241 irregular and potentiophonic words. This yielded a reduction from 12.7% surface errors before the workshop (*SD* 6.5%) to 9.5% (*SD* 5.2%) after it, which approached significance, *t*(6) = 2.26, *p* = 0.06.

Finally, the effect on the reading errors of the participants with vowel letter dyslexia was evaluated via the rate of vowel letter errors (substitutions, additions, migrations, and omissions of vowel letters) out of the 60 non-words they read. This yielded a reduction from 29.3% vowel letter errors [*SD* 23.4%] to 19.4% [*SD* 14.0%], which was, again, non-significant, *t*(5) = 1.70, *p* = 0.15.

### Attention Tests

#### Sustained Attention

**FIGURE 2:**
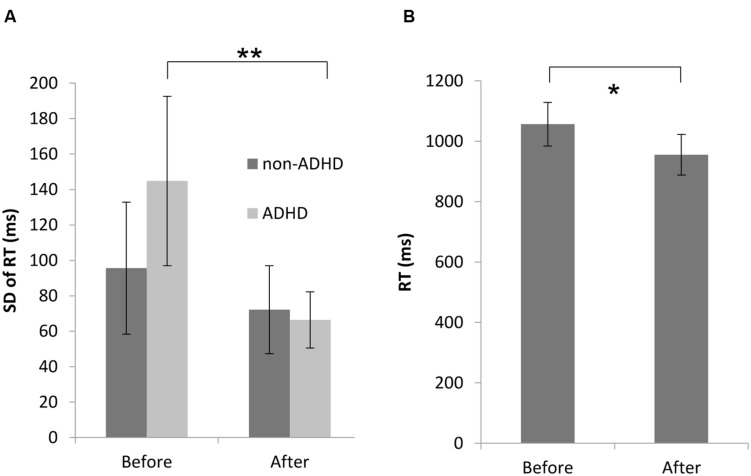
**Improvement in sustained attention among ADHD and non-ADHD participants (A) and in selective attention (B).** ^∗^*p* < 0.01, ^∗∗^*p* < 0.001.

A significant reduction was found in the standard deviations of reaction times (RTs) in the CPT task (before: 117.5 ms [*SD* 50.3], after: 69.5 ms [*SD* 22.2], *t*(17) = 4.37, *p* < 0.001, Cohen’s *d* = 1.29). Overall, 72% of participants (13 out of 18) had larger standard deviations compared to the norm in this measure before the workshop (>77 ms), while only 28% (five participants) performed above the norm after it. Repeated measurements ANOVA for the difference in standard deviations of reaction times between ADHD and non-ADHD participants revealed a significant time (pre–post intervention) by ADHD diagnosis interaction [*F*(1,16) = 9.21, *p* = 0.008, $\eta_{p}^{2}=0.37$]. As can be seen in **Figure 2A**, Tukey HSD *post hoc* comparisons revealed that the workshop had significantly improved ADHD participants’ sustained attention (standard deviations of reaction times) by 54.2% (before: 144.8 ms [*SD* 51.12], after: 66.3 ms [*SD* 17.01], *Tukey HSD*: *p* = 0.0003, Cohen’s *d* = 2.17). The improvement of 24.6% among non-ADHD participants was not statistically significant (before: 95.6 ms [*SD* 39.3], after: 72.1 ms [*SD* 26.2], *Tukey HSD*: *p* = 0.25, Cohen’s *d* = 0.72).

An additional significantly reduction was found in the measure of commissions: the participants had significantly fewer commissions after the workshop (before: 1.9% [*SD* 2.7%], after: 0.4% [*SD* 0.6%], *t*(17) = 2.69, *p* = 0.015, Cohen’s *d* = 0.80).

#### Selective Attention

Two repeated measures ANOVA’s were performed with the within-subjects factors of time (before/after the workshop), target (with/without target) and number of distracters (4, 8, 16, or 32) in the conjunctive search task, one for RTs and one for accuracy. As can be seen in **Figure 2B**, a significant reduction after the workshop was found in RTs (before: 1002.6 ms [*SE* 65.3], after: 898.9 ms [*SE* 60.1], *F*(1,17) = 7.53, *p* = 0.01, $\eta_{p}^{2}=0.31$). No significant interactions with time were obtained, and the change in accuracy after the workshop was non-significant (before: 95.9% [*SE* 1.0%], after: 97.7% [*SE* 0.8%], *F*(1,17) = 2.39, *p* = 0.14). No significant differences were obtained for the search slope, *t*(17) = 0.83, *p* = 0.42).

#### Orienting of Attention

Two repeated measures ANOVAs were performed with the within-subjects factors of time (before/after the workshop) and target (valid/invalid cue) in the peripheral cueing paradigm, one for RTs and one for accuracy. A significant reduction was found in RTs (before: 757.4 ms [*SE* 54.9], after: 632.6 ms [*SE* 44.9], *F*(1,16) = 10.55, *p* = 0.005, $\eta_{p}^{2}=0.40$). In addition, a significant overall improvement in accuracy was obtained (average of valid and invalid trials, before: 92.1% [*SE* 1.9%], after 96.4% [*SE* 0.9%], [*F*(1,16) = 5.31, *p* = 0.035, $\eta_{p}^{2}=0.25$]. The difference between valid and invalid conditions did not change significantly between before and after the workshop, *t*(16) = 0.27, *p* = 0.79.

#### Executive Attention

Two repeated measures ANOVAs were performed with the within-subjects factors of time (before/after the workshop), kind of task (location/direction), and congruency (congruent/incongruent) in the location-direction Stroop-like task. A significant reduction in RTs (overall location and congruity) was obtained (before: 708.1 ms [*SE* 48.4], after: 605.0 ms [*SE* 43.03], *F*(1,15) = 6.81, *p* = 0.02, $\eta_{p}^{2}=0.31$). In addition, a significant overall improvement in accuracy was obtained (before: 95.0% [*SE* 1.1%], after: 97.5% [*SE* 0.6%], *F*(1,15) = 4.53, *p* = 0.05, $\eta_{p}^{2}=0.23$). The difference between the interference effect before and after the workshop was not significant, *t*(15) = 1.83, *p* = 0.09.

### Psychological Measures

The means and standard deviations of the participants’ scores on the various questionnaires are presented in **Table 5.** Paired *t*-tests revealed significant improvements in most of the assessed dimensions. Following the workshop, there was an increase in participants’ self-reported mindfulness (*p* < 0.001), and a decrease in their perceived stress (*p* < 0.001), rumination, depression, and sleep disturbances (*p*’s < 0.05).

**Table 5:** Scores of psychological measures among MBSR participants, before and after the workshop, and the *t*-tests for dependent samples their *p*-values, and Cohen’s *d* effect sizes comparing before and after.

	Before		After		*n*	*t*	*p*	Cohen’s *d*
	Mean	*SD*	Mean	*SD*				
Mindfulness	2.88	0.68	3.39	0.75	19	–4.28	<0.001	0.98
Perceived stress	2.39	0.76	1.55	0.79	19	4.75	<0.001	1.09
Rumination	3.67	0.92	3.26	0.81	18	2.49	0.02	0.59
Depression	2.12	0.53	1.90	0.52	18	2.46	0.02	0.58
Sleep disturbances	3.07	1.16	2.73	1.16	19	2.21	0.04	0.51
Reflection	3.98	0.71	3.77	0.71	18	1.91	0.07	0.45
State anxiety	2.33	0.64	2.08	0.66	19	1.74	0.10	0.40
Life satisfaction	3.52	1.30	3.83	1.13	18	–1.39	0.18	0.33

*Shaded cells indicate a significant improvement between before and after the MBSR training.*

### Correlations

The overall improvement in reading errors was significantly correlated with the improvement in the rate of commissions in the CPT (*r* = 0.71, *p* = 0.01), and with the improvement in self-reported mindfulness (*r* = –0.71, *p* = 0.009).

Significant correlations were obtained between the improvements in selective attention accuracy and improvement in life satisfaction (*r* = 0.71, *p* = 0.01) and sleep disturbances (*r* = –0.74, *p* = 0.009); between improvements in the cost-benefit task accuracy and improvement in sleep disturbances (*r* = –0.80, *p* = 0.003); and between reduction in commissions in the CPT task and improvement in rumination (*r* = 0.74, *p* = 0.009) and life satisfaction (*r* = –0.77, *p* = 0.005).

## Discussion

This study aimed at evaluating the effects of mindfulness meditation on reading, attention, and psychological measures in adults with reading and/or attention impairments. The main question regarded the nature of the relationships within the triangle of mindfulness meditation, attention, and specific functions of the reading process. Mindfulness practice was found to improve the reading of the dyslexic participants, as expressed by a general reduction in their reading errors rate. Importantly, this effect stemmed from a specific effect of mindfulness that encouraged reading via the lexical route, leading to a reduction in errors resulting from reading by grapheme-to-phoneme conversion, especially for the participants with ADHD.

In the domain of attention functions, mindfulness practice was found to reduce impulsivity and enhance sustained attention, as well as shorten reaction times to tasks measuring selective, sustained, executive, and orienting of attention functions. Self-reports indicated that the participants also felt improvement in most of the psychological domains that were evaluated, most prominently in mindfulness and perceived stress. Significant correlations indicated that the reading improvement was related to a decrease in impulsivity (fewer commissions in the CPT test). Additional significant correlations were found between psychological and attention changes following the workshop.

Possibly the most important finding of this study, and one that sheds light on the nature of the effect of mindfulness practice on reading, is the specific effect mindfulness had on errors. Reading can proceed via two different routes: a lexical route, which employs knowledge of words, where reading proceeds via identification of the whole word in the orthographic lexicon, which then activates the phonological output lexicon. The other route is a sublexical route, where words are read via grapheme-to-phoneme conversion. The lexical route is the more accurate, efficient and rapid route, whereas reading words via the sublexical route is slow, and often leads to inaccurate reading. For example, reading the word *talk* via the sublexical route may result in pronouncing the *l*, reading it as “talc.” Such reading is phonologically plausible, but is an inaccurate reading of the target word, and this is the error type that benefitted the most from mindfulness practice. Importantly, this applied when we looked at all the participants, not only for the ones who had the relevant type of dyslexia, and the effect was in place mainly for the dyslexic participants who also had attention disorders (all but one of them had impaired sustained attention).

This result suggests that mindfulness practice keeps reading “on the right track” – in this case, the lexical route. Thus, whereas attention disorders as well as temporary inattention may allow diversion onto the sublexical route, mindfulness assists the reader in keeping reading on the mindful route. This finding is also consistent with the finding that the improvement was significant only for words but not for non-words: if the mindfulness workshop affected reading by leading readers to read words via the lexical, rather than the sublexical route, this would not be expected to affect non-words, which are read exclusively via the sublexical route.

In line with our hypothesis, the MBSR workshop was also found to improve the functioning of sustained-, selective-, orienting-, and executive attention functions, similar to previous findings (e.g., Jha et al., 2007; Zylowska et al., 2008; Jensen et al., 2012; Morrison et al., 2014). The improvement in attentional functioning was expressed via decreased reaction times in all four functions. Importantly, MBSR had a specific effect on sustained attention beyond response times: it significantly reduced commission errors, which are indicative of impulsivity, and it reduced the variation in reaction times in the CPT task, indicating improved sustained attention. In fact, more than half of the participants who performed above the norm before the intervention performed normally after it. Additionally, faster responses of participants were not accompanied by decreased accuracy rates (in fact, accuracy in the orienting of attention and executive attention even significantly improved), thus providing evidence for a more efficient attentional performance.

The picture that emerges with respect to the relation between attention and reading is quite intricate. Firstly, the results of the current study not only indicate that MBSR helped our dyslexic participants who had sustained attention disorder to stay on the lexical route; they also show that MBSR did not have a specific effect on any type of dyslexia, nor did it have an effect on any specific error type beyond surface errors. These results join previous studies that have shown the independence of dyslexias and attention: Lukov et al. (2015) showed double dissociations between various types of dyslexia and each of the attention functions: they reported individuals with LPD who did not have any attentional deficit, as well as individuals with attentional deficit who did not have LPD; similarly, they reported double dissociations between attentional dyslexia and attention; between vowel dyslexia and attention; between phonological buffer dyslexia and attention; between neglect dyslexia at the word level and attention; and between surface dyslexia and attention. Overall, it seems these dyslexias do not stem from attention disorders. Additionally, drugs that help (some) individuals with attention disorders do not reduce reading errors in dyslexia. Lukov and Friedmann (2007) and Keidar and Friedmann (2011) tested individuals with dyslexia and ADHD whose attention disorders were relieved with methylphenidate. Their reading errors were not affected by methylphenidate, supporting the independence between reading and attention disorders. Finally, reports of dyslexias that selectively affect the reading of words and not of numbers or other signs (c.f., Friedmann and Nachman-Katz, 2004; Friedmann et al., 2010a; Collis et al., 2013) further support the point that general attention cannot underlie dyslexia.

The current study thus adds a piece to the puzzle, by clarifying that whereas attention problems do not underlie dyslexia, they may allow the diversion of reading to the sublexical route, which, in turn, may result in inaccurate reading – surface errors. This aspect of the relation between (sustained)^3^^3^ attention and reading is the one that the current study suggests can be amended via mindfulness meditation, which helps the reader stay on the right, lexical track.

It should be noted that reading via the sublexical route not only causes slow and inaccurate reading, but it may also affect reading comprehension: when the word *now*, for example, is read via the sublexical route, it stands the risk of being read like “know,” and hence also comprehended as such. The same with homophones, such as *which, write*, and, for some speakers, also *route* (and root). Reading comprehension is, no doubt, an orchestrated effort of various cognitive and linguistic skills, including lexical, syntactic, and semantic abilities, as well as decoding, motivation, and many others (Wasserman, 2012; Szterman and Friedmann, 2014). We may cautiously suggest that one of the sources of the effect of attention on reading comprehension (e.g., Solan et al., 2003; Kieffer et al., 2013) may be the effect of attention on the use of the right route (or the write root).

The increased ability to stay on the lexical track was correlated with self-reported mindfulness. The attentional aspect that most likely led to the increased ability to stay on the lexical track, and which mediated the effect of mindfulness on reading, is the reduction of impulsivity (as measured by the number of commission errors in the CPT task). The overall reduction in reading errors was significantly correlated with the reduction of commissions in the CPT task, and the reduction of reading errors was mainly present in the dyslexic participants who also had a sustained attention deficit. The mediating role of sustained attention in improving reading performance is consistent with Lam and Beale (1991), who claimed that poor reading comprehension is partly a result of poor sustained attention.

The reduction in surface errors and the improvement in attention measures cannot be ascribed to training effects that result from administering the same tests twice, 3 months apart. We administered the exact same tests twice, 3 months apart, to participants with similar characteristics of dyslexia and attention who did not participate in a meditation workshop, and they showed no improvement in reading error rates or in attention measures.

Consistent with previous reports (e.g., Kabat-Zinn et al., 1992), MBSR was also found to have significant positive effects on the participants’ psychological wellbeing, including the relief of stress and depression. These benefits are also important to the application of mindfulness for individuals with dyslexia or ADHD, as it may successfully address some of the psychological distress experienced by many of them. To date, most interventions are either built on the training of a cognitive deficit that presumably underlies the reading deficiency (e.g., phonology based-training, Shaywitz et al., 2002; Simos et al., 2002), or designed to match the individual’s specific dyslexia type. Examples of the latter include tracking letters with the index finger for LPD (Friedmann and Rahamim, 2014), using colored lines or blinking lights to the left of the word for neglect dyslexia (Nachman-Katz and Friedmann, 2010), or using a word-sized window for attentional dyslexia (Ellis and Young, 1996; Shvimer et al., 2009; Friedmann et al., 2010b). Despite the usefulness of existing interventions, none of them address other domains of difficulty experienced by many dyslexic individuals, such as inattention, heightened stress, and negative views of the self. The advantage of mindfulness practice is that, beyond keeping the readers on the lexical track, it also helps related cognitive and affective processes over the long term.

The psychological improvements related to MBSR were found to be correlated with attentional measures. The strongest correlation was between the improvement in orienting of attention and the reduction of sleep disturbances. In agreement with McCarthy and Waters (1997), Sanders and Reitsma (1982), and Mander et al. (2008) who found sleep deprivation to cause impairments in orienting of attention, it is suggested that the observed reduction in sleep disturbances found among participants following the mindfulness practice contributed to enhanced functioning of this attention system.

This study is the first to show that mindfulness training can improve reading aloud of single words. This improvement in reading following mindfulness practice is in accordance with the results of Mrazek et al. (2013), who demonstrated an improvement in GRE reading comprehension scores among healthy participants after eight mindfulness meetings of 45 min four times a week for 2 weeks. The positive effect of mindfulness practice on individuals with reading and/or attention problems opens promising new directions for intervention in these populations. It should be noted, however, that our observations rely on a small and heterogeneous sample, and as such, should be treated as preliminary.

## Conclusion

Our study provides support to the idea that mindfulness-based interventions can be used to significantly improve reading as well as the quality of life of individuals with dyslexia and ADHD. The effectiveness of this technique, as well as its simplicity, offer these individuals a new hope for addressing their experienced difficulties, with suggested long-term effects. It also sheds important light on the intricate relation between attention and reading, suggesting that mindfulness assists readers in staying on the lexical track for reading.

## Author Contributions

NF, ZB, and RT conceived the idea for the study and the research questions. RT was in charge of the meditation practice. NF created the reading tasks, tested the reading of the participants (together with Rakefet Keidar, whom we thank very much) and analyzed the reading data (together with Lilach Khentov-Kraus, and Liora Lopez Morsian, whom we wholeheartedly thank). ZB ran the attention and psychological tests before and after the workshop as part of her MA thesis. Mainly RT, but also ZB and NF, did the statistical analyses. RT and NF wrote together the final manuscript.

## Conflict of Interest Statement

The authors declare that the research was conducted in the absence of any commercial or financial relationships that could be construed as a potential conflict of interest.

## Footnotes

1 Additional types of dyslexia exist, but we present here the types that were mostrelevant for the current study. For a fuller exposition of dyslexia types, please seeColtheart and Kohnen (2012) and ?

2 www.tfasim.org.il

3 Most of the participants with attention disorders and dyslexia in our study had asustained attention impairment. The finding that the main effect of keeping thereader on the lexical route happened with these participants, and the significantcorrelations between improved reading and reduced commissions suggest thatlexical reading was modulated by reduced impulsivity and improved performancein the CPT task. Cestnick and Coltheart (1999) discussed a different aspect of relationbetween reading and attention: the need for orienting of visual–spatial attentionin reading via grapheme-to-phoneme conversion. It might be that people who arespecifically affected by orienting of attention deficit would show a different picturethan the participants in the current study, with improved non-word reading.

## APPENDIX

### Appendix A: Further Reading Tests to Establish the Diagnosis of the Dyslexia Type

To establish the diagnosis of each type of dyslexia, we further ran tests of reading aloud as well as lexical decision and written word comprehension, with stimuli sensitive to each type of dyslexia. In these additional tests, reading aloud was done without time limit, and the participants were requested to read aloud as accurately and as quickly as possible, and the first responses were counted, even when they were later self-corrected. In the lexical decision and the comprehension tasks, the participants were requested to perform the tasks in silent reading, without sounding out the words they read.

The results of each participant in each of the further reading tasks was compared to those of age-matched controls. In the reading aloud tasks, the number of errors of each type (reading via the sublexical route, vowel omission, substitution, addition, migration, consonant omission, substitution, addition, migration, migrations between words, voicing errors, semantic errors) was compared to the number of these errors in the control group. In the lexical decision and comprehension tasks, the percentage of correct responses was compared to that of the age-matched controls.

#### Letter Position Dyslexia

To establish the diagnosis of letter position dyslexia, which is characterized by letter migrations within words, we used, beyond the oral reading tasks of migratable words described above, also tasks that tested the participants’ silent reading of words that are most sensitive to this dyslexia– migratable words.

Additional tasks involved **same-different decision** in which the participant was presented with 60 word pairs, half of which differed in middle letter order (clam–calm), and was requested to determine whether the words in the pair are same or different; **lexical decision task**, in which the participants saw 60 items, half of them words and half migratable non-words (pecnil) and were requested to determine whether the item was a word; and a **reading comprehension task** that included 50 triads. Each triad consisted of a target migratable word, and two words to choose from: one word that is semantically associated with the target word, and one that is semantically associated with a word that can result from a transposition of middle letter (dairy → milk, notebook). The participants were requested to circle the word that is semantically associated with the target word.

#### Attentional Dyslexia

To establish the diagnosis of attentional dyslexia, characterized by migrations of letters between neighboring words (and by omissions of an instance of a letter that appears in two neighboring words in the same position), beyond the two word-pair reading tests, the participants read aloud an additional list of non-word pairs.

The **migratable non-word** **pair** list included 30 3-letter non-word pairs in which letter migration between words would result in existing words. A large number of migration errors between words in these tasks indicates that the reader has attentional dyslexia.

#### Surface Dyslexia

Each participant with suspected surface dyslexia was tested, in addition to the screening test and the potentiophone reading task, with a lexical discrimination task of pseudo-homophones, and a homophone comprehension task.

##### Pseudo-homophone Lexical Decision

The lexical decision task contained 66 word pairs. Each pair included a word spelled correctly and its pseudo-homophone (e.g., knife-nife). For each pair, the participants were requested to circle the word that was spelled correctly.

##### Homophone–Potentiophone Reading Comprehension

The reading comprehension task included 40 triads. Each triad consisted of a target word, and two words to choose from: one word that is semantically associated to the target word, and a homophone or a potentiophone of the associated word (e.g., bottle – bear beer). The participants were requested to circle the word that is semantically associated with the target word.

#### Vowel Dyslexia

To establish the diagnosis of vowel dyslexia, characterized by substitutions, omissions, additions, and migrations of vowel letters, the participants read in addition to the non-word lists, another word list, and performed two additional tasks of lexical decision and word comprehension.

#### Reading Words

The word list included 100 words, 3- to 8-letter long (mean = 4.17, *SD* 0.56). Words in this list were selected so that each word had at least two lexical options for vowel letter errors, namely, at least two vowel errors made in each target word create other existing words. (For example, a relevant word in English would be the word “form,” which can be read with a vowel migration as *from*, with a vowel substitution as *firm* or *farm*, and with a vowel addition as *forum*.)

#### Lexical Decision

The vowel dyslexia lexical decision task contained 80 items: 45 non-words in which a vowel error creates existing Hebrew words and 35 existing words – 16 of which included a vowel letter and 19 without vowel letters. The items in the task were 2–8 letters (*M* = 4.8, *SD* 1.13). The participants were requested to silently read each word and to circle the words that exist in Hebrew.

#### Reading Comprehension

The reading comprehension task for vowel dyslexia included 52 triads. Each triad consisted of a target word (3–6 letters long, *M* = 4.4, *SD* 0.75), and two to four words to choose from: one word that is semantically associated with the target word, and the rest are words that are semantically associated with words that can result from a vowel error in the target word (form → shape, to, ranch). The participants were requested to circle the word that is semantically associated with the target word.
